# Predicting mortality among septic patients presenting to the emergency department–a cross sectional analysis using machine learning

**DOI:** 10.1186/s12873-021-00475-7

**Published:** 2021-07-12

**Authors:** Adam Karlsson, Willem Stassen, Amy Loutfi, Ulrika Wallgren, Eric Larsson, Lisa Kurland

**Affiliations:** 1 Department of Medical Sciences, Örebro University, Örebro, Sweden; 2 Division of emergency Medicine, University of Cape Town, Cape Town, South Africa; 3 AASS Research Centre, Department of Science and Technology, Örebro university, Örebro, Sweden; 4 Department of Clinical Science and Education, Karolinska Institutet, Söderssjukhuset, Stockholm, Sweden; 5 Departmen of Emergency Medicine, Örebro University Hospital and School of Medicine, Örebro University , Örebro, Sweden; 6 Department of Infectious Diseases, Centralsjukhuset, Karlstad, Sweden; 7 Department of Medical Sciences, Örebro University, Södra Grev Rosengatan 30, 703 62 Örebro, Sweden

**Keywords:** Assessment, Clinical assessment, Emergency care systems, Emergency department, Infectious diseases

## Abstract

**Background:**

Sepsis is a life-threatening condition, causing almost one fifth of all deaths worldwide. The aim of the current study was to identify variables predictive of 7- and 30-day mortality among variables reflective of the presentation of septic patients arriving to the emergency department (ED) using machine learning.

**Methods:**

Retrospective cross-sectional design, including all patients arriving to the ED at Södersjukhuset in Sweden during 2013 and discharged with an International Classification of Diseases (ICD)-10 code corresponding to sepsis. All predictions were made using a Balanced Random Forest Classifier and 91 variables reflecting ED presentation. An exhaustive search was used to remove unnecessary variables in the final model. A 10-fold cross validation was performed and the accuracy was described using the mean value of the following: AUC, sensitivity, specificity, PPV, NPV, positive LR and negative LR.

**Results:**

The study population included 445 septic patients, randomised to a training (*n* = 356, 80%) and a validation set (*n* = 89, 20%). The six most important variables for predicting 7-day mortality were: “fever”, “abnormal verbal response”, “low saturation”, “arrival by emergency medical services (EMS)”, “abnormal behaviour or level of consciousness” and “chills”. The model including these variables had an AUC of 0.83 (95% CI: 0.80–0.86). The final model predicting 30-day mortality used similar six variables, however, including “breathing difficulties” instead of “abnormal behaviour or level of consciousness”. This model achieved an AUC = 0.80 (CI 95%, 0.78–0.82).

**Conclusions:**

The results suggest that six specific variables were predictive of 7- and 30-day mortality with good accuracy which suggests that these symptoms, observations and mode of arrival may be important components to include along with vital signs in a future prediction tool of mortality among septic patients presenting to the ED. In addition, the Random Forests appears to be a suitable machine learning method on which to build future studies.

**Supplementary Information:**

The online version contains supplementary material available at 10.1186/s12873-021-00475-7.

## Introduction

Sepsis is defined as “life-threatening organ dysfunction caused by a dysregulated host response to infection” [1] and has an annual incidence of approximately 840/100000 in Sweden [2] and the incidence is increasing [3]. Sepsis mortality is high and caused almost one in five deaths worldwide in 2017 [4]. Improving our understanding of the importance of the clinical presentation of septic patients could enable early identification of patients likely to have a poor outcome [5]. This is relevant since early treatment with antibiotics and fluid resuscitation has been shown to reduce mortality [6, 7].

The current sepsis criteria are based on the Sequential Organ Failure Assessment (SOFA) score which are selected, at least in part, so as to include patients with an infection with a predicted poor outcome; measured as a mortality of approximately 10% [1]. Several scoring tools have been suggested for predicting sepsis mortality in the emergency department (ED) setting [8, 9]. However, these scoring tools, are based on vital signs and have a limited accuracy [8, 9]. Vital signs alone are, however, insufficient predictors, as one in five patients with severe infection have normal vital signs in the ED. [10] Thus, illustrating the need of an approach other than using vital signs to identify septic patients at risk of poor outcome.

We therefore chose to include measures of the presentation of the septic patients to the emergency department using machine learning. With increased computer power, together with the ability of self-learning and the capability to handle big data, different machine learning models have come to be used in the health care system with promising results [11]. Interestingly, sepsis mortality has previously been shown to be able to be predicted by using the Random Forest, a machine learning method [12]. This demonstrates that machine learning methods can be beneficial for improving the prediction accuracy.

Variables reflective of symptom presentation have been shown to be predictive of mortality among septic patients [13, 14]. Although the prior study using machine learning methods did include a large number of variables [12] none of them were reflective of symptoms at presentation. Therefore, the aim of the current study was to identify variables predictive of 7- and 30-day mortality among septic patients presenting to the ED based on the clinical presentation using machine learning.

## Methods

### Study design and setting

This was a retrospective cross-sectional study using previously identified variables [15] reflective of the presentation of septic patients arriving to the ED at Södersjukhuset. The hospital is located in Stockholm, and has more than 120,000 annual ED visits [16]. The study period was between January 1st 2013 and December 31st 2013.

### Study population

The inclusion criteria were patients ≥18 years of age, admitted to in-hospital care via the ED at Södersjukhuset and discharged from in-hospital care with an International Classification of Disease, Tenth Revision, (ICD-10) code corresponding to sepsis (A02.1, A22.7, A26.7, A32.7, A39.2, A39.4, A40.0 – A40.3, A48 - A49, A41.0 - A41.5, A41.8 - A41.9, A42.7, B37.7, R57.2, R65.0–65.1).

The exclusion criteria were healthcare-associated infection (HCAI), defined as sepsis onset after 48 h from arrival to the ED, [17] patients arriving by emergency medical services (EMS) with ongoing treatment for sepsis or other infectious diseases, unknown mode of arrival and the lack of personal identification number and medical record.

### Definitions and predictive variables

Sepsis was defined as discharge from in-hospital care with an ICD-10 code corresponding to sepsis as specified above. Data was collected when the SEPSIS-2 criteria were in use [18]. The study population included both EMS patients, arriving by ambulance or helicopter, and non-EMS patients, including all other means of arrival to the ED. The definition of severe sepsis was in accordance with a prior definition adapted for emergency care [19].

A total of 90 previously identified variables reflecting the clinical presentation of septic patients to the ED (i.e. vital signs, symptoms, observations and information from medical history, see Supplementary figure 1) were used, [15] in addition to mode of arrival. I.e. a total of 91 variables were included and used as input for the machine learning methods, as described below.

### Ethical approval and consent to participate

Ethical approval was obtained from the regional review board (“Regionala Etikprövningsnämnden i Stockholm”) in Stockholm, diary number 2012/1288.31/3 and 2015/1019–32. All methods were carried out in accordance with relevant guidelines and regulation. Informed consent was waivered by the regional review board in Stockholm as the current study was retrospective and based on a review of medical records.

### Statistical method

#### Descriptive statistics

IBM SPSS Statistics for Macintosh, version 26.0 (IBM corp., Armonk, N.Y., USA) was used for the descriptive analysis, i.e. calculating mean, median, confidence interval and interquartile range for the characteristics of the study population. Shapiro-Wilks test was used to test for normality.

#### Balanced random forests

The supervised machine learning models were developed using the Balanced Random Forest Classifier from the Imblearn collection [20]. This method can be used to build prediction models and to identify associations between specific variables and predicted outcome in unbalanced data. The Balanced Random Forest Classifier technique approaches the challenge of an imbalanced dataset by under-sampling the majority class (bootstrapping) and applying ensemble learning [21]. Thus, the class distribution is changed in order to represent classes equally in each tree; in this case “Patients who died within 7 or 30 days” and “Patients who survived”. Prior study has shown that under-sampling is a more effective method to balance data compared to over-sampling, [22] explaining the choice of using under-sampling in the current study. A 10-fold cross validation was implemented with an 80 to 20 percentage train:test distribution. In each fold, the Balanced Random Forest Classifier included 100 decision trees. Each decision tree was created from a randomly selected subset of the fold’s training set through bootstrapping and included an equal number of patients who died and survived. The ensemble learning method called feature bagging was implemented in the development of each tree, randomly selecting a subset of variables, equal in size to the square root of all variables, to be tested in each node split. When the model makes a prediction, it is based on the majority vote from each of the 100 decision trees. The fold’s test set was used to determine the accuracy, described as area under the ROC curve (AUC), sensitivity, specificity, positive predictive value (PPV), negative predictive value (NPV), positive likelihood ratio (LR) and negative LR. The mean value of the accuracy from all 10 folds is presented in the results. The SHapley Additive explanation (SHAP) interpreter was used to illustrate the relationship between specific predictive variables and the outcome. An exhaustive search was made describing how the mean AUC changes depended on the number of variables included to determine the number of variables to include in the final model. For each iteration of the exhaustive search, the least important variable, described in Gini Impurity, was excluded. See Supplementary figure 2 and 3 for details.

## Results

**Fig. 1:**
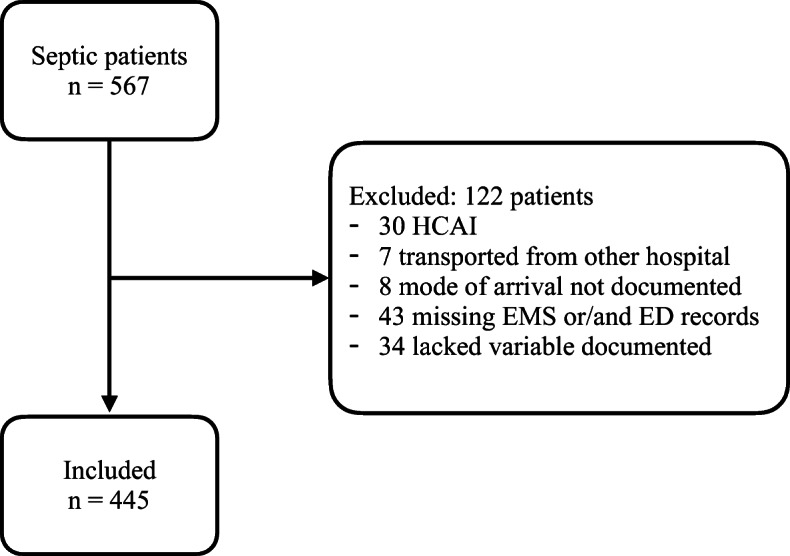
Flow chart of inclusion and exclusion criteria. HCAI = Health care associated infection, EMS = Emergency Medical Services, ED = Emergency Department

In accordance with inclusion and exclusion criteria, a total of 445 patients were enrolled in the current study. See Fig. 1.

The median age was 73 years (IQR 59–84) and 52.6% were men. A total of 323 patients arrived to the ED via EMS and the median length of in-hospital stay was 4 days (IQR 2–9). A total of 63 (14.2%) patients died within 7 days and 98 (22.0%) patients died within 30 days. See Table 1 for characteristics of the study population**.**

**Table 1:** Characteristics of the study population

Variable	Total (*n* = 445)
Median age (IQR)	73.0 (59.0–84.0)
Male gender (%)	234 (52.6)
Severe sepsis (%)	221 (49.7)
Length of hospital stay (IQR)	4.0 (2.0–9.0)
In-hospital mortality (%)	83 (18.7)
1-day mortality (%)	29 (6.5)
7-day mortality (%)	63 (14.2)
30-day mortality (%)	98 (22.0)
1 year mortality (%)	175 (39.3)
RETTS priority	
- Red (%)	159 (35.7)
- Orange (%)	130 (29.2)
- Yellow (%)	116 (26.1)
- Green (%)	24 (5.4)
- Blue (%)	4 (0.9)

19 patients lacked information regarding severe sepsis and 12 patients lacked information regarding RETTS priority. *IQR=* Interquartile range, EMS = Emergency medical service, *RETTS=* Rapid Emergency Triage and Treatment System

### Predicting 7-day mortality

The accuracy of the model predicting 7-day mortality did not improve by including more than six variables, see Supplementary figure 2. These six most important variables for predicting 7-day mortality were in descending order: “fever”, “abnormal verbal response”, “low oxygen saturation”, “arrival by EMS”, “abnormal behaviour or level of consciousness” and “chills”. See Table 2 for the prevalence of these variables in the study population.

**Table 2:** Prevalence of the most important variables for 7-day mortality

Variable	Patients died within 7 days (%) *n* = 63	Patients survived 7 days (%) *n* = 382
Abnormal behaviour or level of consciousness	24 (38.1)	63 (16.5)
Abnormal verbal response	25 (39.7)	55 (14.4)
Arrival by EMS	59 (93.7)	264 (69.1)
Chills	2 (3.2)	91 (23.8)
Fever	18 (28.6)	234 (61.3)
Low oxygen saturation	20 (31.8)	35 (9.2)

*EMS=* Emergency medical service

**Fig. 2:**
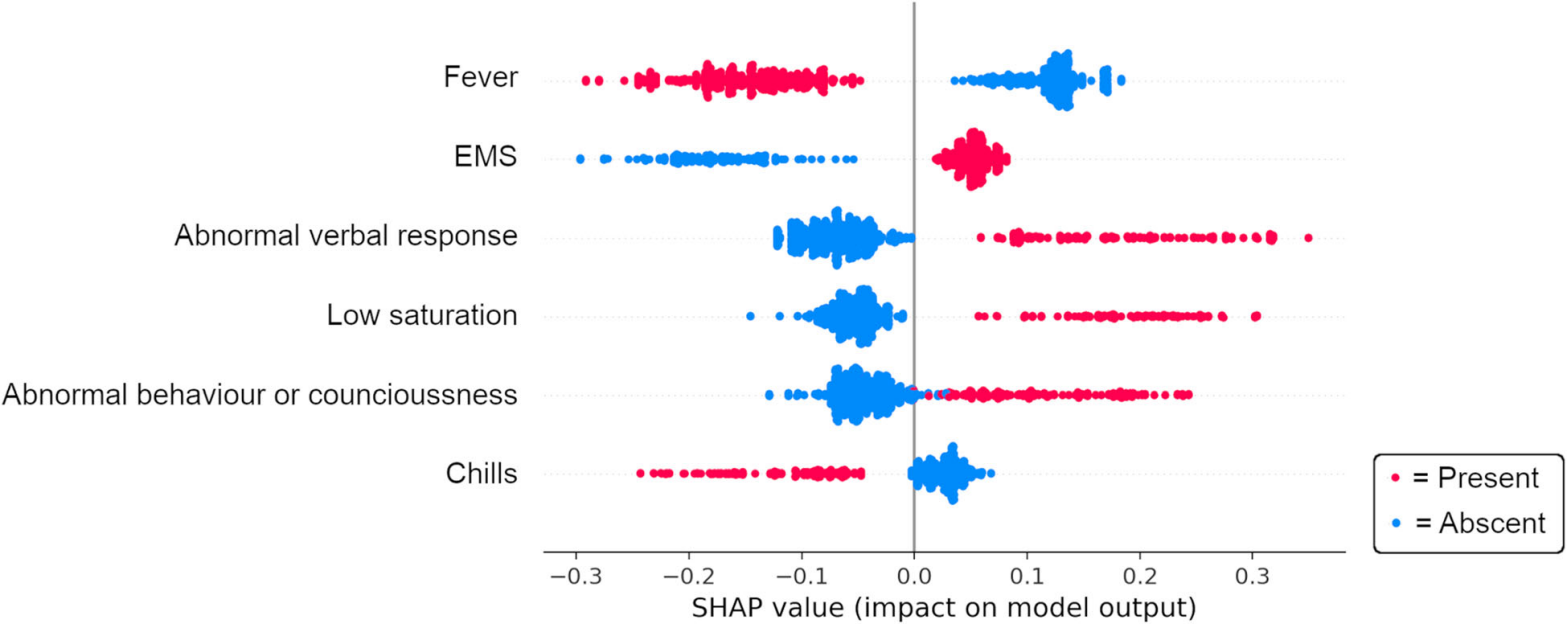
SHAP-summary plot describing the relationship between the six most important variables and 7-day mortality. Each dot corresponds to one patient in the testing population and represents all ten folds. EMS = Emergency medical service

When only these six variables are included, the Balanced Random Forest achieved a sensitivity of 0.84 (CI 95%, 0.78–0.89), specificity 0.67 (CI 95%, 0.64–0.70), PPV 0.31 (CI 95%, 0.28–0.33), NPV 0.96 (CI 95%, 0.95–0.97), positive LR 2.61 (CI 95%, 2.32–2.90) and negative LR 0.24 (CI 95%, 0.16–0.33). A ROC curve was calculated and AUC = 0.83 (CI 95%, 0.80–0.86), see Supplementary Figure 4. To illustrate the relationship between these six most important variables and 7-day mortality, see Fig. 2.

### Predicting 30-day mortality

The accuracy of the model predicting 30-day mortality did not improve by including more than six variables, see Supplementary figure 3. These six most important variables for predicting 30-day mortality were in descending order: “abnormal verbal response”, “fever”, “chills”, “arrival by EMS”, “low oxygen saturation” and “breathing difficulties”. See Table 3 for the prevalence of these variables in the study population.

**Table 3:** Prevalence of the most important variables for 30-day mortality

Variable	Patients died within 30 days (%) *n* = 98	Patients survived 30 days (%) *n* = 347
Abnormal verbal response	37 (37.8)	43 (12.4)
Arrival by EMS	88 (89.8)	235 (67.7)
Breathing difficulties	32 (32.7)	63 (18.2)
Chills	2 (2.0)	91 (26.2)
Fever	33 (33.7)	219 (63.1)
Low oxygen saturation	26 (26.5)	29 (8.4)

*EMS=* Emergency medical service

**Fig. 3:**
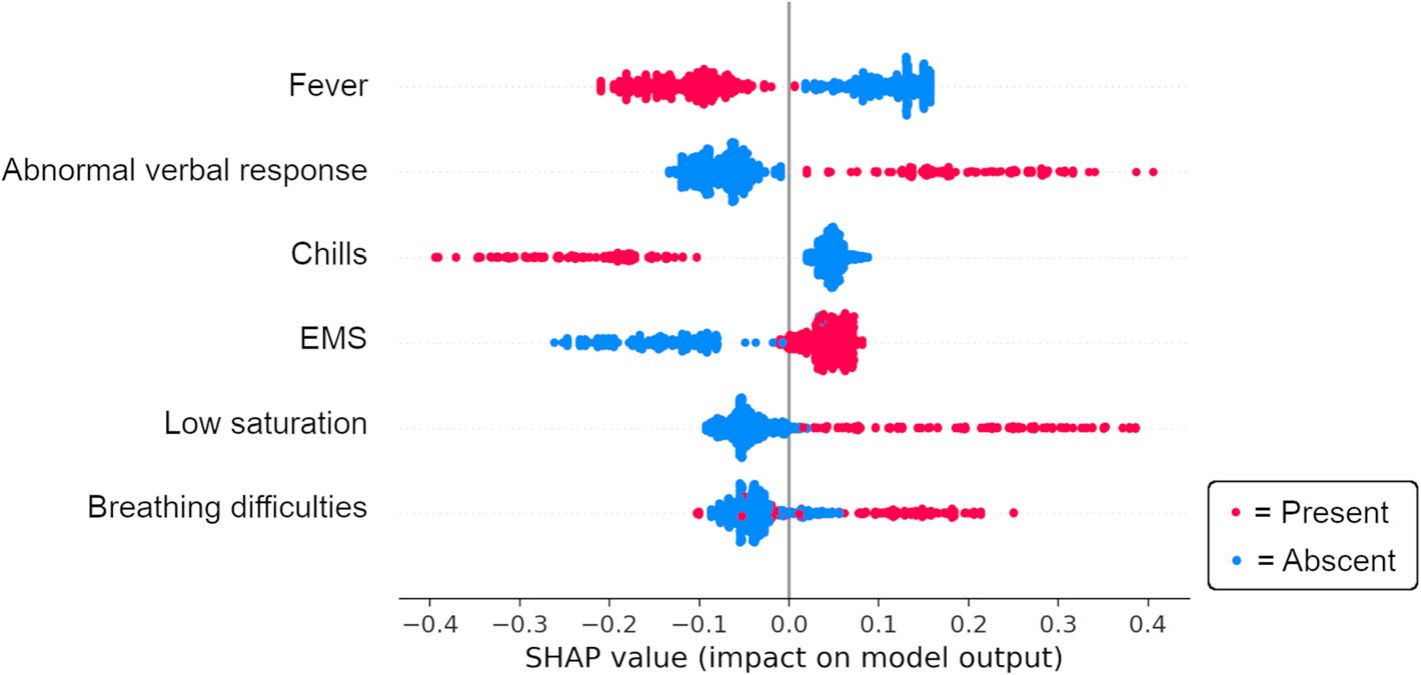
SHAP-summary plot describing the relationship between the six most important variables and 30-day mortality. Each dot corresponds to one patient in the testing population and represents all ten folds. EMS = Emergency medical service

When only these six variables are included, the Balanced Random Forest achieved a sensitivity of 0.87 (CI 95%, 0.81–0.93), specificity 0.64 (CI 95%, 0.61–0.67), PPV 0.41 (CI 95%, 0.39–0.44), NPV 0.95 (CI 95%, 0.92–0.97), positive LR 2.45 (CI 95%, 2.22–2.68) and negative LR 0.20 (CI 95%, 0.11–0.30). A ROC curve was calculated and AUC = 0.80 (CI 95%, 0.78–0.82), see Supplementary figure 5. To illustrate the relationship between the six most important variables and 30-day mortality, see Fig. 3.

A sub-analysis was made using the five most important variables and replacing the variable breathing difficulties with tachypnea. A ROC curve was calculated and AUC = 0.77 (CI 95%, 0.75–0.79), see Supplementary figure 6.

## Discussion

The results show that the following six variables: “fever”, “abnormal verbal response”, “low oxygen saturation”, “arrival by EMS”, “abnormal behaviour or level of consciousness” and “chills” were the most important variables for predicting 7-day mortality among septic patients presenting to the ED. The variables “abnormal verbal response”, “fever”, “chills”, “arrival by EMS”, “low oxygen saturation” and “breathing difficulties” were the most important variables for predicting 30-day mortality. We suggest the current study be viewed as a proof-of-principle that machine learning, specifically Random Forest, is a suitable tool with which to include a large number of variables reflecting the presentation of sepsis.

It is interesting that the model when using mode of arrival, abnormal verbal response, abnormal behaviour or level of consciousness, chills and two vital signs (low saturation and fever) could predict 7-day mortality with a good AUC and sensitivity. Several of these variables are not included in previously suggested predictive tools [8, 9, 12]. Prior studies support that low oxygen saturation, abnormal level of consciousness, the absence of chills, fever and rigors in addition to hyperglycaemia, are predictive of poor outcome for septic patients [13, 14]. It may be surprising that the results suggest that fever and chills are protective in sepsis, ie have a lower mortality. However, we speculate that fever and chills can represent a well-functioning immune response. Another explanation to a better outcome could be that patients with these symptoms are more easily recognised as septic, hence receive timely treatment. Prior studies included either septic patients arriving with EMS or patients with septic shock, [13, 14] while the current study includes all septic patients presenting to the ED-setting. Thus, is likely to include septic patients with a greater variation of disease severity. To our knowledge, there is no prior study including this number of variables reflecting the presentation in the association between clinical presentation to the ED and sepsis mortality.

Overall, the accuracy was similar for the model predicting 30-day mortality as that for the 7-day mortality. It is, however, notable that the model predicting 30-day mortality included “breathing difficulties” and not “abnormal behaviour or level of consciousness”. Furthermore, no change was seen regarding the models performance when breathing difficulties was replaced with tachypnea. Thus, showing that symptoms and signs related to breathing are important variables when predicting 30-day mortality for septic patients. Considering both models, we suggest that the model predicting 7-day mortality is more clinically relevant for ED practitioners as knowledge regarding short-term outcome could be used for bedside clinical decision-making in the ED.

The clinical presentation of sepsis is often non-specific and variable [15]. We suggest that this makes the application of machine learning suitable, due to its ability to identify associations not previously considered among a large number of variables and that its potential could be increased in larger data sets. The Random Forest Classifier has been applied with promising results in several fields such as forecasting gross domestic product growth [23] and urban planning, [24] and also predicting sepsis mortality [12]. Thereby, illustrating that the six most important variables identified in the current study could be included to support disposition decisions and predicting the likelihood of poor outcome which may have an impact on resource utilization and allocation. However, more importantly, we believe the current study to be proof-of-principle of the possibilities of the method. The current study is not without limitations. First of all, the data was collected when SEPSIS-2 criteria were in use [18]. Some of the septic patients included in the current study may not be classified as septic in accordance with SEPSIS-3 [1]. However, sepsis according to SEPSIS-3 is more prevalent than severe sepsis according to sepsis-2 criteria [2] and almost half of the current study population had severe sepsis. Therefore, it is reasonable to assume that a large proportion of the current study population would be septic also in accordance with SEPSIS-3. The use of ICD-10 codes to define sepsis may also be questioned. Although it is previously described that only one in seven of septic patients are identified when using ICD-10 codes, [25] it is however likely that these are the sickest septic patients receiving ICD 10 codes relating to sepsis, which is also supported by the large proportion of patients with severe sepsis. In addition, identifying septic patients based on ICD-10 codes is a common method in registry studies [26]. Considering the use of the Random Forest, each decision tree is trained on bagged data using random selection of features, therefore gaining a full understanding of the decision process is difficult. However, with the implementation of SHAP interpreter, it is possible to illustrate the association between specific predictive variables and the outcome. Random forest was chosen as it is a tree-based model, which follows a decision pattern clinically used in emergency medicine and controls for the over-fitting otherwise observed in other tree-based models. The decision to use six variables in the final model is to a certain extent arbitrary. However, the AUC did not increase by including more variables, as presented in Supplementary figure 2 and 3. From a low- or middle-income country’s perspective, where IT-systems may not be available to the same extent, one could argue that fewer variables are better as this simplifies the use of a predictive model. Despite the limitations of the current study, the results indicate that there is an association between specific variables measurable in the ED and sepsis mortality. Further studies are needed to evaluate the use of these specific six variables in models or tools predictive of outcome, but more importantly to use the Random Forest and other machine learning methods to enable the analysis of a large amount of data to build predictive models.

## Conclusion

The results indicate that the following variables, measurable in the ED, were predictive of both 7-day and 30-day mortality among septic patients: fever, abnormal verbal response, low saturation, arrival by EMS and chills. Moreover, abnormal behaviour or level of consciousness was predictive of 7-day mortality, while breathing difficulties was more important for predictive 30-day mortality. These results suggest that symptoms, observations and mode of arrival may be important variables to include in a future prediction tools of mortality among patients with suspected sepsis. These results do, however, need to be validated in other cohorts. In addition, the Random Forests appears to be a suitable machine learning method on which to build future studies.

## Supplementary Information

**Additional file 1: Supplementary Figure S1.** Table describing all 91 variables included.

**Additional file 2: Supplementary Figure S2.** Predicting 7-day mortality - Mean AUC compared with number of variables included.

**Additional file 3: Supplementary Figure S3.** Predicting 30-day mortality - Mean AUC compared with number of variables included.

**Additional file 4: Supplementary Figure S4.** The receiver operating characteristic curve for predicting 7-day mortality.

**Additional file 5: Supplementary Figure S5.** The receiver operating characteristic curve for predicting 30-day mortality.

**Additional file 6: Supplementary Figure S6.** Sub-analysis for predicting 30-day mortality - the receiver operating characteristic curve. The predictive variable breathing difficulties is replaced with tachypnea.

## Data Availability

The datasets used and/or analysed during the current study are available from the corresponding author on reasonable request. The costume code used for the statistical analyses can be found in the following DOI link: 10.5281/zenodo.4642088

## Abbreviations

ED: Emergency department
ICD: International classification of diseases
AUC: Area under the ROC curve
PPV: Positive predictive value
NPV: Negative predictive value
LR: Likelihood ratio
SHAP: SHapley Additive explanation
EMS: Emergency medical services
SOFA: Sequential organ failure assessment
HCAI: Healthcare-associated infection
IQR: Interquartile range
