# Supplementary material for: Predicting mortality among septic patients presenting to the emergency department–a cross sectional analysis using machine learning
Source: BMC Emerg Med. 2021 Jul 12;21:84. doi: 10.1186/s12873-021-00475-7 (PMC8276466; doi:10.1186/s12873-021-00475-7)
Supplement: Supplementary file 1 — Additional file 1: Supplementary Figure S1. Table describing all 91 variables included. [file 12873_2021_475_MOESM1_ESM.pdf]

Variables n = 91	Vital signs n = 7	Symptoms n = 35	Observations n = 38	Other variables n = 11
Abdominal pain		X		
Abnormal micturition, defined as haematuria without trauma, bad smelling or cloudy urine, increased frequency of urination		X		
Abnormal behaviour or level of consciousness, excluding abnormal verbal response			X	
Abnormal verbal response, defined as no/decreased/changed verbal response			X	
Airway secretion, including expectorations, crackles and similar expressions			X	
Anxiety or fear		X		
Arrival by EMS				X
Back pain		X		
Blisters, on the skin			X	
Bloodstained patient			X	
Breathing difficulties, statement difficulties to breath, dyspnea, shortness of breath, shallow breathing or similar expressions		X		

Bruises or petechiae		X	
Cardiac-arrest		X	
Change of skin turgor		X	
Chills	X		
Chest pain	X		
Chronically compromised breathing, such as painful conditions or neurological diseases compromising breathing			X
Compromised immune system, Chemotherapy or other immunosuppressive treatment			X
Costovertebral angle tenderness		X	
Cough	X		
Current antibiotic treatment			X
Cyanosis, including blue fingernails/lips/toes		X	

Decreased ability to stand or walk, including need to carry/lift the patient	X		
Decreased general condition, including expressions such as poor general condition, affected general condition	X		
Decreased miscellaneous mobility, including expressions such as stiffness when trying to move arms, disability to sit or disability to squeeze the investigators hand	X		
Diarrhea	X		
Dizziness	X		
Dry mucous membranes, of the mouth		X	
Dysarthria, slurred speech (but non-affected level of consciousness)	X		
Dysfunction of urinary catheters			X
Extremity pain	X		
Exuding skin		X	
Fallen		X	

Feeling of depression		X		
Fever, statement fever or statement temperature > 38° Celsius	X*			
Focal neurological findings			X	
Found on the floor, or corresponding place			X	
Gastrointestinal bleeding, including melena, hematemesis, haematochezia		X		
General pain		X		
Headache		X		
High blood sugar, plasma glucose > 12 mmol/L, regardless diabetes or not				X
High blood pressure, statement high blood pressure or statement blood pressure ≥ 160 mmHg systolic or ≥ 100 mmHg diastolic	X			
High CRP, taken previous to ED arrival				X
History of positive findings in blood culture, positive blood culture taken previous to EMS arrival during a visit to the hospital or by other health care provider but the patient is now at home.				X

Hypothermia, defined as statement hypothermia or “very low temp” or statement temperature < 36° C.	X			
Icterus			X	
Irregular pulse			X	
Joint pain		X		
Known ongoing or recent infection				X
Low blood pressure, statement systolic blood pressure ≤ 90 mmHg.	X			
Loss of energy, defined as fatigue, weakness, faintness or similar expressions		X		
Low oxygen saturation, defined as statement oxygen saturation <90 %	X			
Malaise, defined as expression such as felling sick, feeling bad, not feeling well and similar expressions		X		
Mottling			X	
Nausea		X		
Non-measurable breathing variables			X	

Non-measurable circulatory variables		X
Obstipation	X	
Obstructive breathing		X
Oedema/swelling		X
Painful muscle cramp	X	
Pale		X
Pale stool		X
Palpitations	X	
Peripheral coldness		X
Photosensitivity	X	
Prolonged capillary refill time		X
Rash on skin		X

Redness of skin		X	
Reduced amount of stool		X*	
Reduced intake of food, fluids or oral medicines, including reduced/no appetite		X*	
Reduced urinary volume		X*	
Recent invasive procedures, including IV drug abuse, surgical and urological procedures, new IV or urinary catheters			X
Remained lying or sitting, statement of being remained sitting or lying in an abnormal way.		X	
Seizure		X	
Sensitivity to sound	X		
Soiled patient, patient wetted from his/her own urine or stool.		X	
Substance abuse, defined as drug abuse, alcohol overconsumption and all other terms indicating substance abyss such as “lives in a home for addicts”, “patient at an outdoor clinic for substance abuse”.			X
Suspected fever, defined as statement feeling hot/warm, increasing temperature or similar expressions.	X		

Sweaty			X
Syncope		X	
Tachycardia, statement heart rate >90/min or expressions sich as rapid heart rate, rapid pulse or similar expressions.	X		
Tachypnea, statement Tachypnea, rapid breathing, high respiratory rate, respiratory rate > 20, or similar expressions.	X		
Temporal deterioration, stated deterioration or expressions describing temporal change			X
Throat pain		X	
Urinary tract pain		X	
Undefined pain		X	
Vomiting		X	
Weak pulse, including difficulties to palpate the pulse			X
Wound pain		X	
Wound or wound infection			X

*Variable could be included in several subgroups. For more details, see Wallgren et al.[15]
