# Supplementary figures and images for: Predicting mortality among septic patients presenting to the emergency department–a cross sectional analysis using machine learning
Source: BMC Emerg Med. 2021 Jul 12;21:84. doi: 10.1186/s12873-021-00475-7 (PMC8276466; doi:10.1186/s12873-021-00475-7)

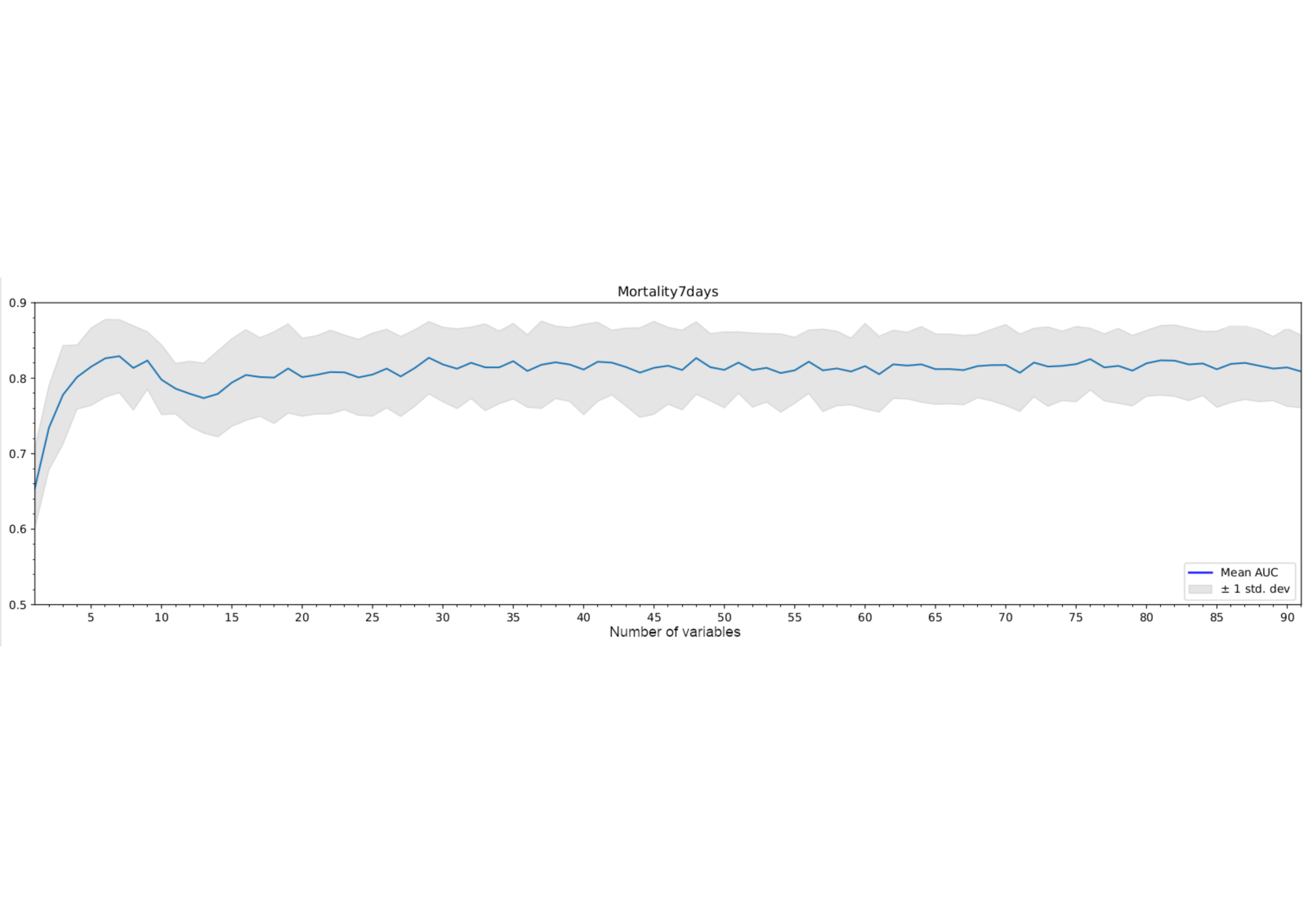

Supplement: Supplementary file 2 — Additional file 2: Supplementary Figure S2. Predicting 7-day mortality - Mean AUC compared with number of variables included. [file 12873_2021_475_MOESM2_ESM.png]

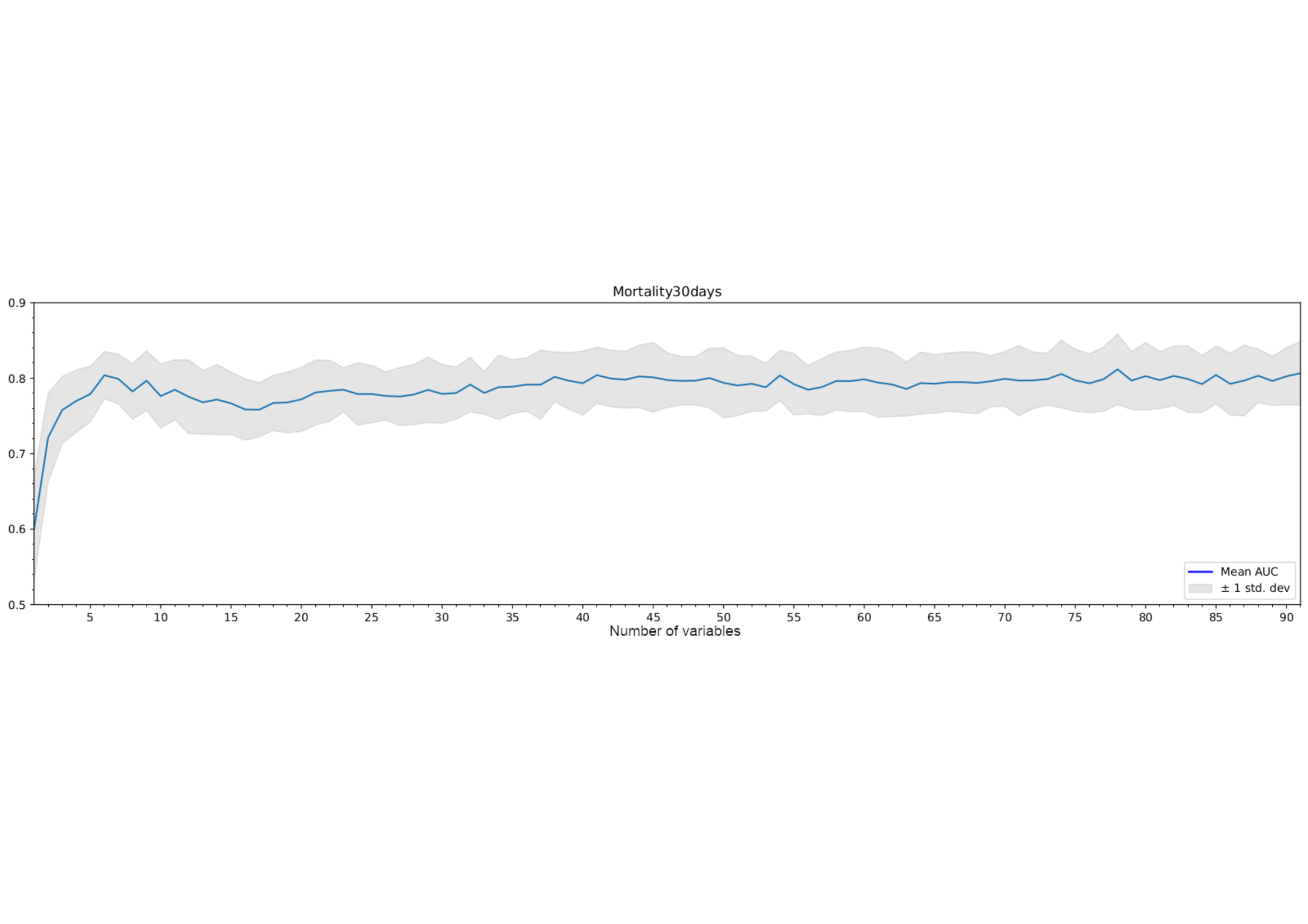

Supplement: Supplementary file 3 — Additional file 3: Supplementary Figure S3. Predicting 30-day mortality - Mean AUC compared with number of variables included. [file 12873_2021_475_MOESM3_ESM.png]

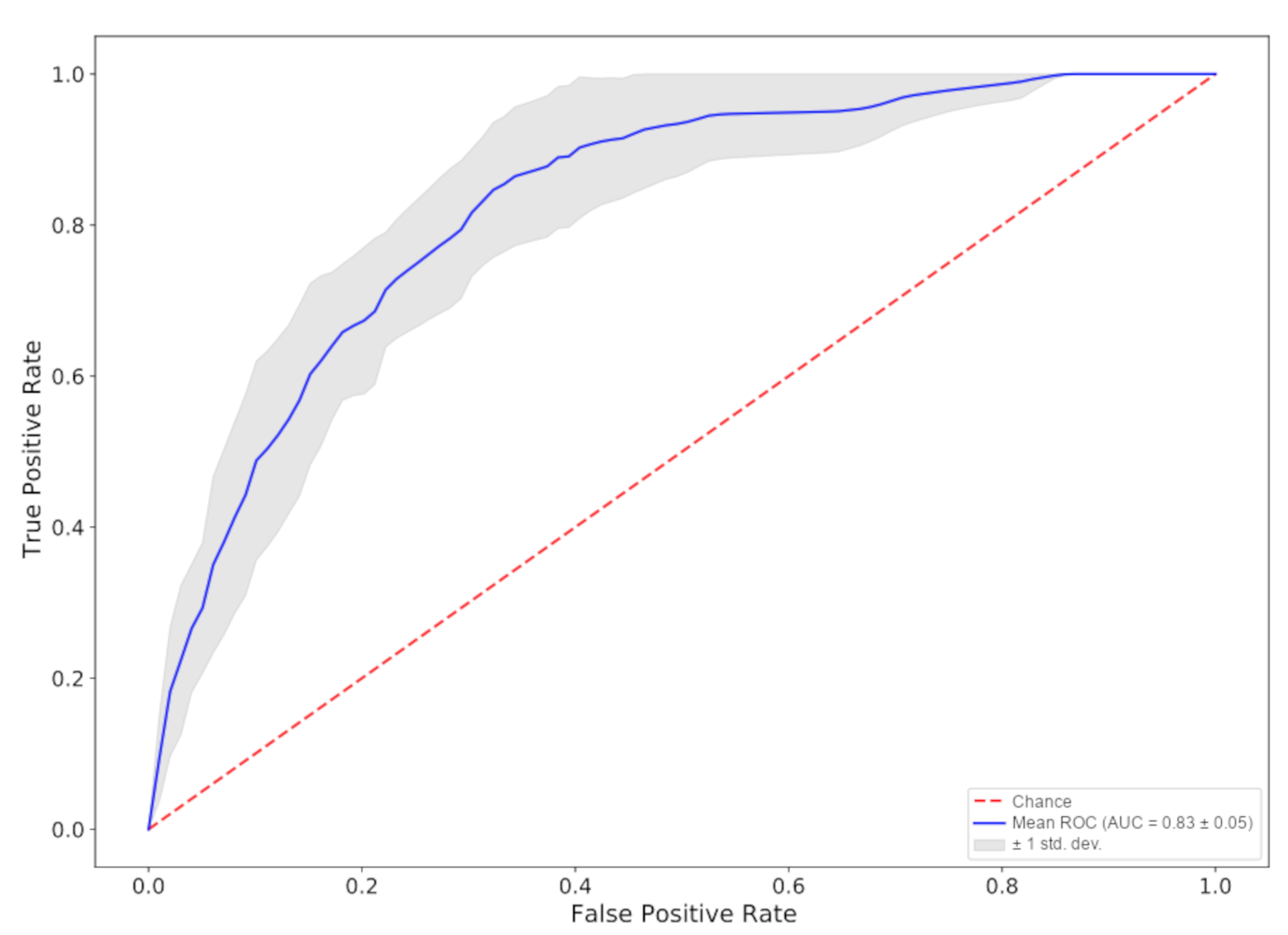

Supplement: Supplementary file 4 — Additional file 4: Supplementary Figure S4. The receiver operating characteristic curve for predicting 7-day mortality. [file 12873_2021_475_MOESM4_ESM.png]

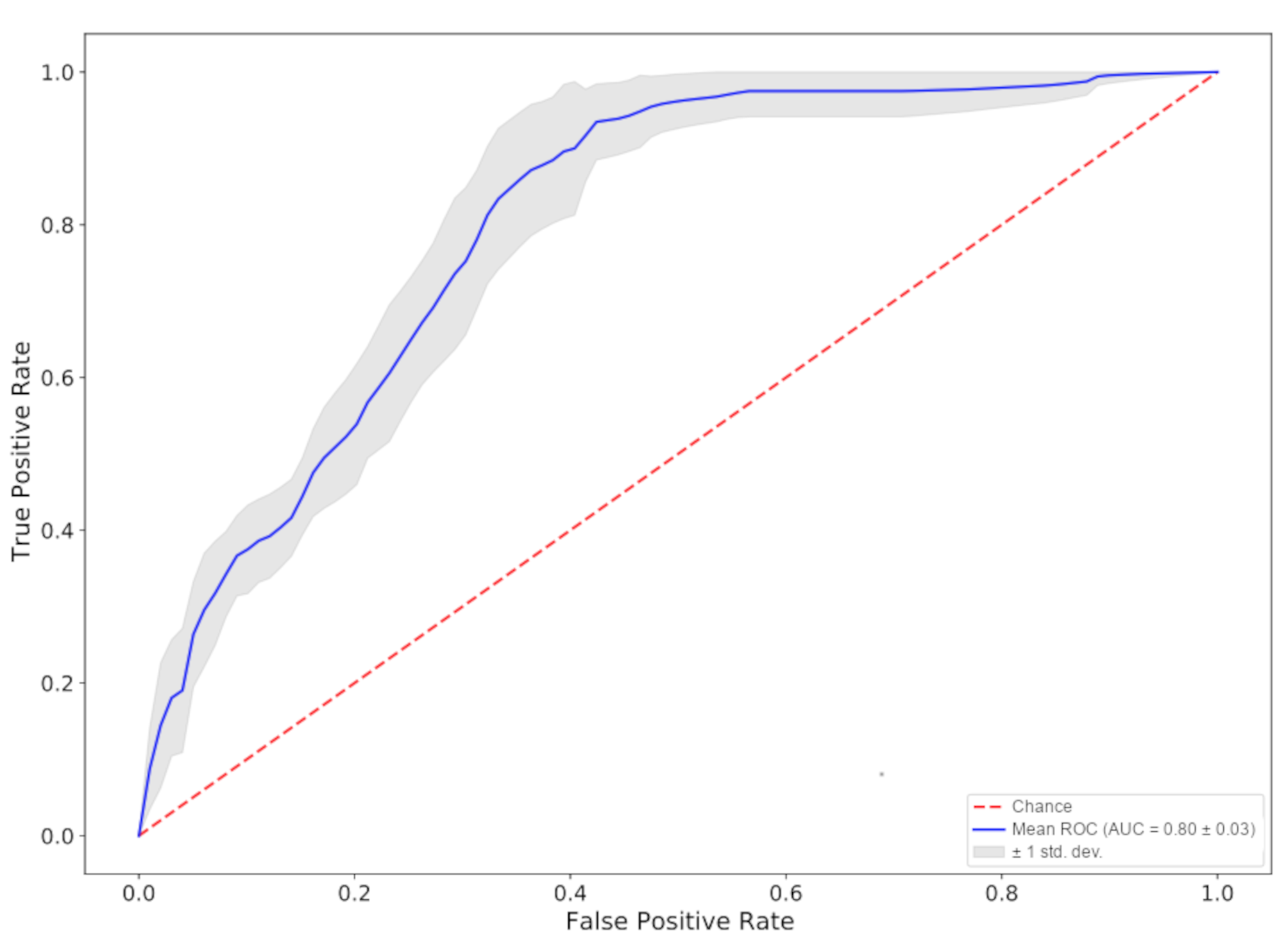

Supplement: Supplementary file 5 — Additional file 5: Supplementary Figure S5. The receiver operating characteristic curve for predicting 30-day mortality. [file 12873_2021_475_MOESM5_ESM.png]

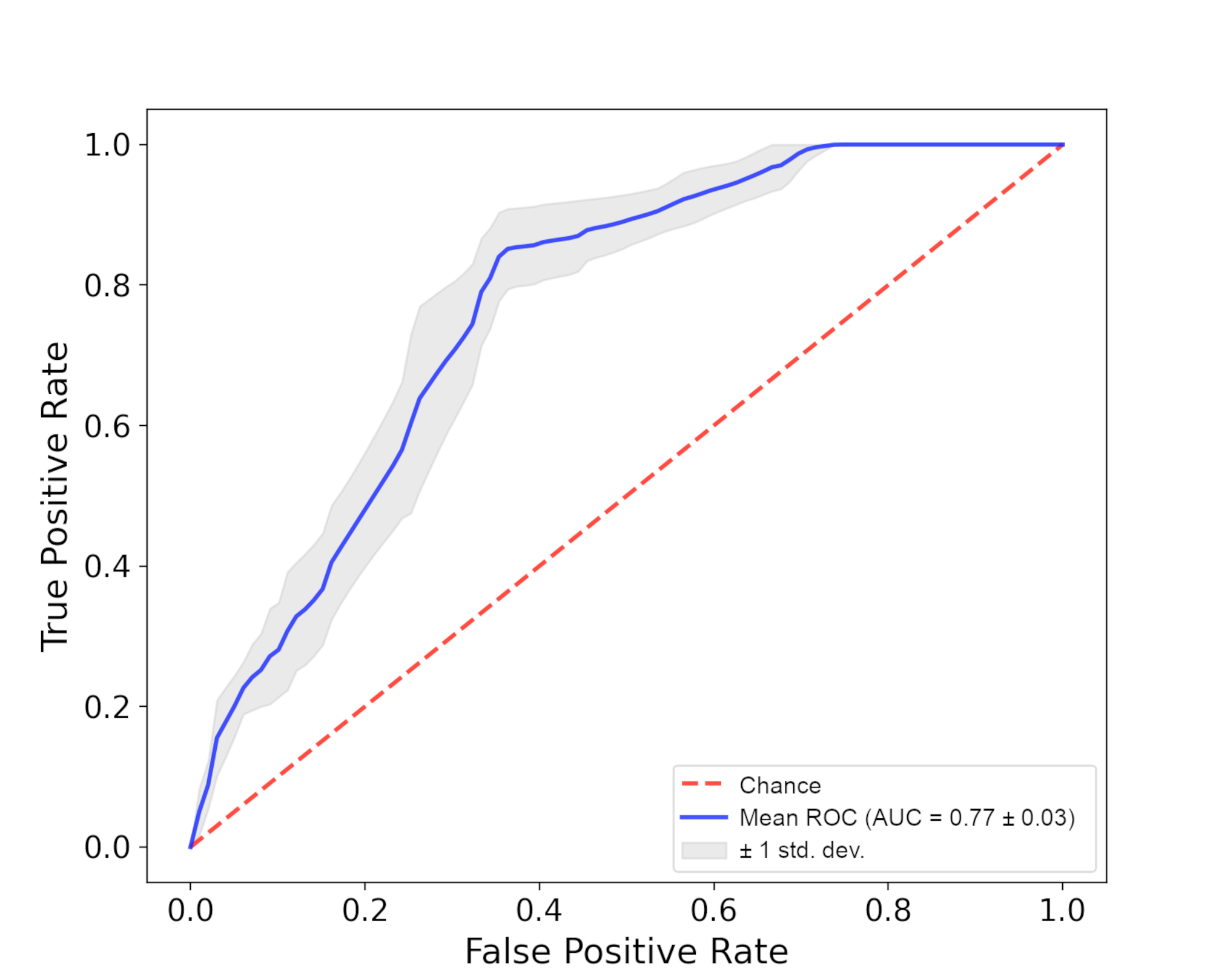

Supplement: Supplementary file 6 — Additional file 6: Supplementary Figure S6. Sub-analysis for predicting 30-day mortality - the receiver operating characteristic curve. The predictive variable breathing difficulties is replaced with tachypnea. [file 12873_2021_475_MOESM6_ESM.png]
